# Initiatives, Concepts, and Implementation Practices of FAIR (Findable, Accessible, Interoperable, and Reusable) Data Principles in Health Data Stewardship Practice: Protocol for a Scoping Review

**DOI:** 10.2196/22505

**Published:** 2021-02-02

**Authors:** Esther Thea Inau, Jean Sack, Dagmar Waltemath, Atinkut Alamirrew Zeleke

**Affiliations:** 1 Medical Informatics Institute for Community Medicine University Medicine Greifswald Greifswald Germany; 2 International Health Department Johns Hopkins Bloomberg School of Public Health Baltimore, MD United States

**Keywords:** data stewardship, FAIR data principles, health research, PRISMA, scoping review

## Abstract

**Background:**

Data stewardship is an essential driver of research and clinical practice. Data collection, storage, access, sharing, and analytics are dependent on the proper and consistent use of data management principles among the investigators. Since 2016, the FAIR (findable, accessible, interoperable, and reusable) guiding principles for research data management have been resonating in scientific communities. Enabling data to be findable, accessible, interoperable, and reusable is currently believed to strengthen data sharing, reduce duplicated efforts, and move toward harmonization of data from heterogeneous unconnected data silos. FAIR initiatives and implementation trends are rising in different facets of scientific domains. It is important to understand the concepts and implementation practices of the FAIR data principles as applied to human health data by studying the flourishing initiatives and implementation lessons relevant to improved health research, particularly for data sharing during the coronavirus pandemic.

**Objective:**

This paper aims to conduct a scoping review to identify concepts, approaches, implementation experiences, and lessons learned in FAIR initiatives in the health data domain.

**Methods:**

The Arksey and O’Malley stage-based methodological framework for scoping reviews will be used for this review. PubMed, Web of Science, and Google Scholar will be searched to access relevant primary and grey publications. Articles written in English and published from 2014 onwards with FAIR principle concepts or practices in the health domain will be included. Duplication among the 3 data sources will be removed using a reference management software. The articles will then be exported to a systematic review management software. At least two independent authors will review the eligibility of each article based on defined inclusion and exclusion criteria. A pretested charting tool will be used to extract relevant information from the full-text papers. Qualitative thematic synthesis analysis methods will be employed by coding and developing themes. Themes will be derived from the research questions and contents in the included papers.

**Results:**

The results will be reported using the PRISMA-ScR (Preferred Reporting Items for Systematic Reviews and Meta-analyses Extension for Scoping Reviews) reporting guidelines. We anticipate finalizing the manuscript for this work in 2021.

**Conclusions:**

We believe comprehensive information about the FAIR data principles, initiatives, implementation practices, and lessons learned in the FAIRification process in the health domain is paramount to supporting both evidence-based clinical practice and research transparency in the era of big data and open research publishing.

**International Registered Report Identifier (IRRID):**

PRR1-10.2196/22505

## Introduction

Advancement in information communication technology is impacting the health ecosystem’s technological and analytical capabilities to store, curate, share, and analyze data from standard and nonstandard sources [1]. In the human health domain, big data may be obtained multidimensionally from records in health care facilities, biomedical research institutions, population surveys, surveillance, and patients [2]. Together, professional data management and big data analytics offer high-potential knowledge to transform health care delivery and life sciences research. The availability of data from numerous sources and advanced analytics promise to improve the prevention, diagnosis, and treatment of diseases and the well-being of individuals and societies [3]. However, health data are often stored in independent noncommunicating silos, where open data sharing remains a challenge [4].

Digitalization brings opportunities and concerns in health care data processing. Despite many potential benefits, it also poses potential threats, such as breaches of privacy, disinformation and misinformation, and cyberattacks [5]. There is a need to balance an individual's rights to the protection of personal data from potential threats with the institutions' needs to process these data. The EU General Data Protection Regulation (GDPR) informs this context. The recent reform in the GDPR focuses on the rights and freedoms of people and the establishment of rules for the processing of personal data [6]. The concerns about privacy and personal data protection resulted in reforms of the existing legislation in the European Union. The GDPR aims to reform the existing measures on the topic of personal data protection of EU citizens with a strong input on the rights and freedoms of people and the establishment of rules for the processing of personal data [7]. OpenEHR is a standard that embodies many principles of interoperable and secure software for electronic health records [8].

The European GDPR is the most recent data regulatory framework as of September 2020 and has implications on the ethical sharing of research data [9]. As the EU population continues to be more conscientious about the data protection regulations for citizens’ sensitive personal data (eg, EU GDPR), patients and the general public are becoming more aware of the use of their personal data [10]. The principle of data minimization implies that personal data shall be adequate, relevant, and limited to only what is necessary in relation to the purposes for which they are processed [7]. 

Boeckhout et al [11] highlighted that the GDPR also ensures that the terms of data use, data subjects, and rights in further processing are clearly defined. It has been suggested that FAIR (findable, accessible, interoperable, and reusable) data and metadata standards could help facilitate compliance with the principle of data minimization by allowing for an assessment of which data to reuse based on an analysis of metadata [11].

Beyan et al [12] have shown that an enormous amount of usable health data is currently imprisoned inside the organizational territories of hospitals, clinics, and within patients’ devices due to ethical concerns and data protection rules. However, data reuse, even if secondary to data collection and first analysis, may drive more extensive and valuable new research directions than intended for the primary purpose [13]. In Germany, for example, the Medical Informatics Initiative aims to use clinical data to improve health research and facilitate the digitalization of medicine on a national scale [14]. France has also launched the Health Data Hub with similar aims [15]. Currently, researchers and stakeholders are working on infrastructure to support distributed and federated solutions to make the data, software, or digital objects smart in their original silos [12]. Europe would benefit from an integrated infrastructure in which data and computing services for big data can be easily shared and reused, and plans are underway to establish the Europe Research Area for this purpose [16]. Finally, funding agencies and open science advocates are insisting on adherence to open science policies and strategies to manage publicly funded research processes and outcomes [17]. The Health Research Board (HRB) of Ireland, for example, has put in place the HRB Policy on Management and Sharing of Research Data, which requires research to be open. This policy is applicable to data gathered and generated in whole or in part from HRB-funded research, starting from January 1, 2020.

The need for good data stewardship among different stakeholders in scientific research is the basis on which the FAIR data principles (findability, accessibility, interoperability, and reusability) were coined in 2014 by the FORCE11 (The Future of Research Communication and e-Scholarship) community [18,19]. These principles were formed to serve as guidance to achieve better research data stewardship practices in the life sciences [20]. They also serve as a set of widely applicable “permissive guidelines,” offering a basis for developing flexible community standards for the health data community [21]. Since research papers and data products are now being recognized as key outcomes of the scientific enterprise, various stakeholders in scientific and governmental institutions are increasing their efforts toward establishing more comprehensive plans for data management and stewardship [16,22]. Adherence to the FAIR principles has been shown to lead to a more transparent approach to data stewardship, which in turn contributes to the maximal use and reuse of data in the scientific community [23]. Consequently, adherence to the FAIR data principles is more frequently expected by researchers, publishers, funding agencies, and policy makers [24]. Achieving data FAIRness also enhances the discovery of, access to, integration of, and analysis of scholarly and scientific data [25].

In 2020, Vesteghem et al [26] outlined data sharing challenges that make data aggregation costlier and more labor intensive in precision oncology. Obstacles include legal issues that hinder data sharing between research groups, privacy issues, ethical issues, data storage issues, and system incompatibility issues [26]. Various initiatives have been launched to tackle these challenges by standardizing and facilitating the implementation of data pipelines [27,28]. Although the application of the FAIR data principles in data stewardship is a fairly new approach in health research, it has been shown to be instrumental in addressing these challenges in the field of precision oncology [14]. It has also been suggested that FAIR data may be useful in addressing the need to generate and share high-quality data to facilitate the World Health Organization elimination goals for neglected tropical diseases [29]. Much work has been conducted to implement the FAIR principles in other domains, such as computational workflows [30], food and nutrition [31], materials science [32], and oceanography [33].

The aims for conducting this work are to (1) provide an overview of applications of the FAIR data principles that are focused on health data research and (2) map out the existing evidence accordingly.

## Methods

### Study Framework

This scoping review will adopt the framework outlined by Arksey and O’Malley [34]. The authors will employ this method to quickly map key concepts underpinning the research area of interest and the main sources and types of evidence available. Our work is focused on an area that we have not seen being reviewed comprehensively. The framework includes the following steps: (1) identifying the research question; (2) identifying relevant studies; (3) selecting the studies; (4) charting the collected data; and (5) collating, summarizing, and reporting the results.

### Stage 1: Identifying the Research Questions

We have already conducted a pilot overview of the existing literature as an informal desk review and literature exploration. This overview included published works in PubMed, Google Scholar, and Web of Science. The medical and public health research librarian used the FAIR data principles’ keywords to match medical subject headings (MeSH) used to tag PubMed peer-reviewed literature, along with combinations of terms used in clinical research, public health, health care, pharmacology, and patient data. Multimedia Appendix 1 enumerates the results of these advanced searches.

As part of the ongoing evidence synthesis from medical and human health research journal articles that used FAIR data markup, the bibliographies of key papers were scrutinized for other complementary publications, and those articles were added to the PubMed collections shared with the authors. Further, as the key FAIR data and health articles inspired new citations, often authored by similar consortia of writers or networks of researchers, the newer citing articles were added to the stage 1 collection to demonstrate possible progress in the field of shared or open medical data. Recurrent alerts were set up to capture newly published literature on PubMed, Google Scholar, and Web of Science (Multimedia Appendix 1). White papers, conference publications, guidelines, and other grey literature from the Google and Web of Science alerts were scrutinized and added to a Dropbox of publications for the principal researchers to review. Close examination of key references in bibliographies and citing articles to gauge the impact of FAIR shared data on ensuing research and health practice will be followed as part of the secondary analysis. Publications from 2020 focusing on open sharing of COVID-19 data will be of particular importance in gauging the impact of the FAIR principles on human health data in pandemics.

Our informal desk review has shown that many approaches used in the implementation of the FAIR data principles are applied to the life sciences domain [18]. We have also seen in the literature that there is indeed a growing interest in following the phases of the research life cycle when conducting research [35,36]. These findings resonate with the authors’ motivation to better understand the approaches used in the implementation of the FAIR data principles and the impact that these implementations may have on the way research in health will be conducted. These findings are also the basis on which the research questions were formulated. As we formulated the research questions, we decided that the review should only include works that show either an actual approach to implementing the FAIR data principles in the health domain or the recorded results of the implementation of the FAIR data principles. The review will exclude works that introduce or give an overview of the FAIR principles. Works that show the implementation of the principles in a domain other than health will also be excluded.

As we intend to conduct this exploratory review in an iterative manner, further refinement of the research questions may become necessary. Close examination of key references in bibliographies and citing articles to gauge the impact of shared data on ensuing research and health practice will be followed as part of the secondary analysis. All proposed refinements of the research questions and search methods will be scrutinized by the authors prior to approval. We will also provide comprehensive provenance information on changes in the protocol to be fully transparent.

### Objectives and Research Questions

The general objective of this protocol is to conduct a scoping review to identify concepts, approaches, implementation experience, and lessons learned from the FAIR data principle initiatives in the health domain. The following research questions (RQs) have been formulated to meet the objective of the scoping review:

- RQ 1: What approaches are being used or piloted in the implementation of the FAIR data principles in the health data domain since the conception of these principles in 2014?
- RQ 2: What are the challenges and risks regarding the approaches used in the practical implementation of the FAIR data principles in the health data domain?
- RQ 3: What are the suggested concepts and approaches to mitigating the concerns of the implementation of the FAIR data principles in the health data domain?
- RQ 4: Which are the active public and private research and service networks involved in the implementation of the FAIR data principles in the health data domain?
- RQ 5: What are the reported outcomes for data sharing, data reuse, and research publication after the implementation of the FAIR data principles in the health data domain?

### Stage 2: Identifying Relevant Studies

With the aid of an experienced research librarian, at least two researchers will identify relevant studies from 3 primary electronic databases: PubMed, Web of Science, and Google Scholar. In addition to those, relevant grey literature from existing networks, relevant organizations, and conferences as well as the reference lists from potential papers will be searched. The keywords for the scoping review search strategies have been categorized tentatively to terms related to the FAIR data principles, data sharing, and health. Although refinement of the selected MeSH ​terms are possible, open terms have been proposed for the construction of the search strategy of this protocol. The Boolean operators “AND” and “OR” will be used to guide the search strategy. The following descriptors and keywords and their combinations were used to construct the strategies: “open science,” “data collection,” “data provenance,” “open access publishing,” “data*,” ”repositor*,“ ”registr*,“ ”pharma*,“ ”health*,“ ”research,“ ”biomedical research,“ ”data management,“ “FAIR data principles,” “FAIR principles,” “FAIR guiding principles,” “Data steward*,” “Data management systems,” “findable,” “findability,” “access,” “accessibility,” “interoperable,” “interoperability,” “reusable,” “reusability” (Multimedia Appendix 1).

The PRISMA-ScR (Preferred Reporting Items for Systematic Reviews and Meta-analyses Extension for Scoping Reviews) reporting guidelines will be used for reporting the findings [37]. The operational definition of “health” for this scoping review is based on the European Union’s 2018 General Data Protection Regulation and the health ecosystems components framed by the World Health Organization [2,6]. Accordingly, health data in this protocol are defined in the context of data from service and research practice in health services (clinical records, electronic health records and electronic medical records, prescribing, diagnostics, laboratory, insurance, disease surveillance, immunization records, public health reporting, vital statistics, registries, clinical trials, clinical research, and public health research).

As an inclusion criterion, we will consider literature published between January 1, 2014, and December 31, 2020. The start date in 2014 is chosen due to the fact that FAIR concept initiatives and official publications became first available in that year. Moreover, to be included as a potential paper, the literature needs to be published in English and include the scope of FAIR principle applications in the health domain (defined by the operational definition). Literature published before 2014, in a language other than English, and in domain areas other than health or the operational definition of health will be excluded. All search results from online databases and grey literature sources will be exported to a reference management software to eliminate duplications. Unique search results will be exported to a screening tool to facilitate an independent screening process for the potential papers.

### Stage 3: Study Selection

Rayyan software (Qatar Computing Research Institute) has been chosen as the primary screening and data extraction tool to expedite the initial screening of abstracts and titles using a semiautomated process while incorporating a high level of usability. This software supports research teams in the easier exploration of literature searches within a shorter time as well as in sharing and comparing individual researchers’ decisions to include or exclude studies [38]. According to the inclusion and exclusion criteria, nonrelevant studies will be excluded from the study at this point. If the relevancy of the publication is unclear from the title or abstract, the reviewer will read the full publication to determine the eligibility of the publication. Any further changes to the search criteria to improve the search findings will be made at this stage as necessary. In the next step, the eligible publications screened in the first stage will be independently read in full by 2 researchers to further determine the relevance of the publication content to the research questions. When agreement cannot be reached during the initial screening and full-text screening stages, an independent researcher will be consulted. A PRISMA flow diagram will be generated to provide visual data for the selection process [37].

### Stage 4: Data Charting

A data-charting form will be used by the reviewers to determine which variables to extract. The form is flexible for continuous updating in an iterative manner during the data-charting process, but any changes will be tracked. The descriptive analytical approach, as described by Arksey and O’Malley [34], will be employed in the data collection process. In this process, the researchers will critically examine the identified articles and documents that meet all of the eligibility criteria and extract the relevant data from each publication using the pretested charting form. The data will be organized into a chart with 2 main sections to describe the overview or summarized basic information of the publication (metadata) and the research questions based on our objectives (Table 1). Initially, 2 authors will independently extract data from the first 5 included studies using the data-charting form and meet to determine whether their approach to data extraction is consistent with the research question and purpose.

**Table 1:** Data-charting form.

Section		Description
**Section 1: Overview**		Summary of the basic information of the publication
	Publication type	Peer reviewed or grey literature
	Country	Name of the country or countries where the study took place or focused on
	Objective	Aim or objective of the publication
	Methodology	The specific procedures or techniques used to identify, select, process, and analyze information
	Study design and data management	Includes whether the researchers used quantitative, qualitative, or mixed-method approaches
	Setting of the study	The site in which the researcher conducted the study
	Summarized results	A short summary of the findings
**Section 2: Research questions**		Includes the research questions and the date that the literature was published
	Suggested health care domain–specific FAIRification^a^ concepts and approaches	A description of FAIRification concepts and approaches in the health care domains
	FAIR implementation challenges, risks, and lessons learned	Encountered challenges or anticipated changes and lessons learned at different stages of FAIR data principle concept introduction, infrastructure implementation, and FAIRness evaluation
	Active networks involved in the implementation of the FAIR data principles in the health domain	Dedicated networks of scientific communities, research institutions, repositories or data archives, consortia, funding agencies, and citizens who are actively engaged in advocating FAIR principle data stewardship in the health care domains
	FAIRification reported outcomes	FAIR implementation outcomes in terms of data sharing, data reuse, and research publication after imposing FAIR data principles in health domain

^a^FAIR: findable, accessible, interoperable, reusable.

### Stage 5: Collating, Summarizing, and Reporting the Results

This scoping review focuses on the range of data curated and the health data research content identified. Quantitative assessment is limited to a count of the number of sources reporting a particular FAIR thematic issue or recommendation. After charting the relevant data from the studies in spreadsheets, the results will be collated and described using summary statistics, charts, figures, and common tools for analytical reinterpretation of the literature [34]. Mapping the themes derived from the research questions (FAIR implementation approaches, available FAIR networks, FAIR infrastructural and security challenges, etc) and other emerging themes during charting and analysis will be done. Moreover, the impact of the findings in relation to the overall study purpose, implications for future research, practice, and policy will be discussed accordingly [34]. The results will be reported using the PRISMA scoping review reporting guidelines [37]. 

## Results

### Overview

Our PubMed preliminary search has yielded 360 results (Multimedia Appendix 1). The search strategy we used to identify these results will be iteratively revised as we search for the results that best fit the inclusion criteria. We are also working on translating this MeSH search strategy into ​terms for alerts on the Google Scholar and Web of Science databases. The identification of relevant studies began in April 2020. Data extraction will be carried out in the last quarter of 2020. After completion of steps 1 to 3, we will use the title and abstract and a full-text review to determine the number of studies that meet the inclusion criteria. Full-text data extraction will also be used to confirm the number of studies included. Step 5 will involve summarizing and synthesizing the results. We anticipate finalizing the manuscript for this work by March 2021.

### Anticipated Outcomes

This scoping review will provide insight on the initiatives, concepts, and implementation practices of FAIR data principles in health data stewardship. More specifically, it will allow for the exploration of (1) approaches being used or piloted for the implementation of the FAIR data principles in the health domain since the conception of these principles in 2014; (2) challenges, risks, lessons learned, and the suggested concepts and approaches to mitigate the concerns of implementation of the FAIR data principles in the health domain; (3) active research and service networks involved in the implementation of the FAIR data principles in the health domain; and (4) the reported outcomes for data sharing, data reuse, and research publication after the implementation of the FAIR data principles in the health domain. We anticipate increases in data repositories demanding FAIR data markup suitable for artificial intelligence extraction of statistics. We also anticipate a greater demand for the implementation of the FAIR principles in light of the ongoing COVID-19 pandemic as well as more open research activities by public and private research and service networks involved in the implementation of the FAIR data principles in the health domain. An example of such an initiative is the Research Data Alliance [39].

The results will be used to generate recommendations on how to integrate the FAIR principles in health research, and we will generate different knowledge dissemination materials to share project results with various stakeholders, partners, associations, and networks who may benefit from this work.

## Discussion

### Future Work

The findings of this proposed work may be used to help identify the types of available evidence that support the incorporation of FAIR data principles in health. The results will also help to clarify key concepts in the scientific literature and serve as an introduction to how research on FAIR practices is conducted. This methodological framework will help us identify the overall state of research activities that explore initiatives, concepts, and implementation practices of FAIR data principles in health data stewardship. The outcome of this review can be used to further determine areas of research based on current gaps in the literature. Conducting this scoping review will also help determine the practicality and relevance of a full systematic review on the same issues by assessing the availability of literature. Similarly, gaps that still exist in the uptake and implementation of the FAIR principles in health research will also be identified as areas of further research. This work will be of interest to various stakeholders, including health and academic institutions, publishers, researchers, and funding agencies. In the wake of the COVID-19 pandemic, it is extremely critical that health data stewardship is practiced in a FAIR manner to facilitate the globally coordinated response [40]. As this work intends to include works that have been published up until December 31, 2020, we expect that we will gather a lot of information about what has been done worldwide regarding the FAIR data principles in health during this critical time. For purposes of the dissemination of the results of this work, the authors will consider submitting abstracts for presentation to various scientific forums and submit a manuscript for publication in a peer-reviewed journal.

### Ethics

Once complete, this work will be published in a peer-reviewed journal, and the results will also be presented at appropriate forums or conferences. Ethical approval is not required, as only secondary data from published sources will be included in the scoping review and the public is not invited to participate in this work.

## Appendix

Multimedia Appendix 1 Supplementary Material.

## Abbreviations

FAIR: findable, accessible, interoperable, and reusable
FORCE11: The Future of Research Communication and e-Scholarship
GPDR: General Data Protection Regulation
HRB: Health Research Board
MeSH: medical subject heading
PRISMA-ScR: Preferred Reporting Items for Systematic Reviews and Meta-analyses Extension for Scoping Reviews
RQ: research question
