# Supplementary material for: Initiatives, Concepts, and Implementation Practices of FAIR (Findable, Accessible, Interoperable, and Reusable) Data Principles in Health Data Stewardship Practice: Protocol for a Scoping Review
Source: JMIR Res Protoc. 2021 Feb 2;10(2):e22505. doi: 10.2196/22505 (PMC7886612; doi:10.2196/22505)
Supplement: Multimedia Appendix 1 [file resprot_v10i2e22505_app1.docx]

## Supplementary Material

Web of Science

| Set |  |
| --- | --- |
| \| #36 \| \| --- \| | (#35 AND #23 AND #14)  *AND* **LANGUAGE:** (English)  Indexes=SCI-EXPANDED, SSCI, A&HCI, CPCI-S, CPCI-SSH, BKCI-S, BKCI-SSH, ESCI, CCR-EXPANDED, IC Timespan=2014-2020 |
| \| # 35 \|  \|  \| \| --- \| --- \| --- \| | #34 OR #33 OR #32 OR 31 OR #30 OR #29 OR #28  Indexes=SCI-EXPANDED, SSCI, A&HCI, CPCI-S, CPCI-SSH, BKCI-S, BKCI-SSH, ESCI, CCR-EXPANDED, IC Timespan=2014-2020 |
| # 34 | \| #27 AND #26 AND #25 AND #24  Indexes=SCI-EXPANDED, SSCI, A&HCI, CPCI-S, CPCI-SSH, BKCI-S, BKCI-SSH, ESCI, CCR-EXPANDED, IC Timespan=2014-2020 \| \| --- \| |
| \| # 33 \|  \|  \| \| --- \| --- \| --- \| | TS="FAIR data" OR AB= "FAIR data"  Indexes=SCI-EXPANDED, SSCI, A&HCI, CPCI-S, CPCI-SSH, BKCI-S, BKCI-SSH, ESCI, CCR-EXPANDED, IC Timespan=2014-2020 |
| # 32 | \| TS=data steward*  Indexes=SCI-EXPANDED, SSCI, A&HCI, CPCI-S, CPCI-SSH, BKCI-S, BKCI-SSH, ESCI, CCR-EXPANDED, IC Timespan=2014-2020 \| \| --- \| |
| # 31 | \| TS="FAIR guiding principles" OR AB="FAIR guiding principles"  Indexes=SCI-EXPANDED, SSCI, A&HCI, CPCI-S, CPCI-SSH, BKCI-S, BKCI-SSH, ESCI, CCR-EXPANDED, IC Timespan=2014-2020 \| \| --- \| |
| # 30 | \| TS="FAIR principles" OR AB="FAIR principles"  Indexes=SCI-EXPANDED, SSCI, A&HCI, CPCI-S, CPCI-SSH, BKCI-S, BKCI-SSH, ESCI, CCR-EXPANDED, IC Timespan=2014-2020 \| \| --- \| |
| # 29 | \|  \|  \| TS="FAIR data principles" OR AB="FAIR data principles"  Indexes=SCI-EXPANDED, SSCI, A&HCI, CPCI-S, CPCI-SSH, BKCI-S, BKCI-SSH, ESCI, CCR-EXPANDED, IC Timespan=2014-2020 \| \| --- \| --- \| --- \| |
| # 28 | \| TI="data management" OR AB="data management"  Indexes=SCI-EXPANDED, SSCI, A&HCI, CPCI-S, CPCI-SSH, BKCI-S, BKCI-SSH, ESCI, CCR-EXPANDED, IC Timespan=2014-2020 \| \| --- \| |
| # 27 | \| TS=interoperable OR TS=interoperability Indexes=SCI-EXPANDED, SSCI, A&HCI, CPCI-S, CPCI-SSH, BKCI-S, BKCI-SSH, ESCI, CCR-EXPANDED, IC Timespan=2014-2020 \| \| --- \| |
| # 26 | \|  \| TS=accessible OR TS=accessibility  Indexes=SCI-EXPANDED, SSCI, A&HCI, CPCI-S, CPCI-SSH, BKCI-S, BKCI-SSH, ESCI, CCR-EXPANDED, IC Timespan=2014-2020 \| \| --- \| --- \| |
| # 25 | \| TS=findable OR TS=findability  Indexes=SCI-EXPANDED, SSCI, A&HCI, CPCI-S, CPCI-SSH, BKCI-S, BKCI-SSH, ESCI, CCR-EXPANDED, IC Timespan=2014-2020 \| \| --- \| |
| # 24 | \| TS=reusability OR TS=reusable  Indexes=SCI-EXPANDED, SSCI, A&HCI, CPCI-S, CPCI-SSH, BKCI-S, BKCI-SSH, ESCI, CCR-EXPANDED, IC Timespan=2014-2020 \| \| --- \| |
| # 23 | \|  \|  \| #22 OR #21 OR #20 OR #19 OR #18 OR #17 OR #16 OR #15 Indexes=SCI-EXPANDED, SSCI, A&HCI, CPCI-S, CPCI-SSH, BKCI-S, BKCI-SSH, ESCI, CCR-EXPANDED, IC Timespan=2014-2020 \| \| --- \| --- \| --- \| |
| # 22 | \|  \| TS=clinical data  Indexes=SCI-EXPANDED, SSCI, A&HCI, CPCI-S, CPCI-SSH, BKCI-S, BKCI-SSH, ESCI, CCR-EXPANDED, IC Timespan=2014-2020 \| \| --- \| --- \| |
| # 21 | \| TS=research data  Indexes=SCI-EXPANDED, SSCI, A&HCI, CPCI-S, CPCI-SSH, BKCI-S, BKCI-SSH, ESCI, CCR-EXPANDED, IC Timespan=2014-2020 \| \| --- \| |
| # 20 | \| TI=biomedical research OR AB=biomedical research  Indexes=SCI-EXPANDED, SSCI, A&HCI, CPCI-S, CPCI-SSH, BKCI-S, BKCI-SSH, ESCI, CCR-EXPANDED, IC Timespan=2014-2020 \| \| --- \| |
| # 19 | \|  \| TS=public health data  Indexes=SCI-EXPANDED, SSCI, A&HCI, CPCI-S, CPCI-SSH, BKCI-S, BKCI-SSH, ESCI, CCR-EXPANDED, IC Timespan=2014-2020 \| \| --- \| --- \| |
| \| # 18 \|  \| \| --- \| --- \| | \| TS=medical data  Indexes=SCI-EXPANDED, SSCI, A&HCI, CPCI-S, CPCI-SSH, BKCI-S, BKCI-SSH, ESCI, CCR-EXPANDED, IC Timespan=2014-2020 \| \| --- \| |
| # 17 | \| TS=pharma* data  Indexes=SCI-EXPANDED, SSCI, A&HCI, CPCI-S, CPCI-SSH, BKCI-S, BKCI-SSH, ESCI, CCR-EXPANDED, IC Timespan=2014-2020 \| \| --- \| |
| \| # 16 \|  \| \| --- \| --- \| | \| TS=health data  Indexes=SCI-EXPANDED, SSCI, A&HCI, CPCI-S, CPCI-SSH, BKCI-S, BKCI-SSH, ESCI, CCR-EXPANDED, IC Timespan=2014-2020 \| \| --- \| |
| # 15 | \|  \|  \| TI=pharma* or AB=pharma*  Indexes=SCI-EXPANDED, SSCI, A&HCI, CPCI-S, CPCI-SSH, BKCI-S, BKCI-SSH, ESCI, CCR-EXPANDED, IC Timespan=2014-2020 \| \| --- \| --- \| --- \| |
| # 14 | \| #13 OR #12 OR #11 OR #10 OR #9 OR #8 OR #7 OR #6 OR #5 OR #4 OR #3 OR #2 OR #1  Indexes=SCI-EXPANDED, SSCI, A&HCI, CPCI-S, CPCI-SSH, BKCI-S, BKCI-SSH, ESCI, CCR-EXPANDED, IC Timespan=2014-2020 \| \| --- \| |
| # 13 | \| TS="open access"  Indexes=SCI-EXPANDED, SSCI, A&HCI, CPCI-S, CPCI-SSH, BKCI-S, BKCI-SSH, ESCI, CCR-EXPANDED, IC Timespan=2014-2020 \| \| --- \| |
| # 12 | \| TS="open publishing"  Indexes=SCI-EXPANDED, SSCI, A&HCI, CPCI-S, CPCI-SSH, BKCI-S, BKCI-SSH, ESCI, CCR-EXPANDED, IC Timespan=2014-2020 \| \| --- \| |
| # 11 | \| TS="data collection"  Indexes=SCI-EXPANDED, SSCI, A&HCI, CPCI-S, CPCI-SSH, BKCI-S, BKCI-SSH, ESCI, CCR-EXPANDED, IC Timespan=2014-2020 \| \| --- \| |
| \| #10 \|  \| \| --- \| --- \| | \| ALL="metadata"  Indexes=SCI-EXPANDED, SSCI, A&HCI, CPCI-S, CPCI-SSH, BKCI-S, BKCI-SSH, ESCI, CCR-EXPANDED, IC Timespan=2014-2020 \| \| --- \| |
| # 9 | \| ALL="data provenance"  Indexes=SCI-EXPANDED, SSCI, A&HCI, CPCI-S, CPCI-SSH, BKCI-S, BKCI-SSH, ESCI, CCR-EXPANDED, IC Timespan=2014-2020 \| \| --- \| |
| # 8 | \|  \|  \| ALL="linked data"  Indexes=SCI-EXPANDED, SSCI, A&HCI, CPCI-S, CPCI-SSH, BKCI-S, BKCI-SSH, ESCI, CCR-EXPANDED, IC Timespan=2014-2020 \| \| --- \| --- \| --- \| |
| # 7 | \| ALL="open data"  Indexes=SCI-EXPANDED, SSCI, A&HCI, CPCI-S, CPCI-SSH, BKCI-S, BKCI-SSH, ESCI, CCR-EXPANDED, IC Timespan=2014-2020 \| \| --- \| |
| # 6 | \| ALL="open repositor*"  Indexes=SCI-EXPANDED, SSCI, A&HCI, CPCI-S, CPCI-SSH, BKCI-S, BKCI-SSH, ESCI, CCR-EXPANDED, IC Timespan=2014-2020 \| \| --- \| |
| # 5 | \| ALL= "data preservation"  Indexes=SCI-EXPANDED, SSCI, A&HCI, CPCI-S, CPCI-SSH, BKCI-S, BKCI-SSH, ESCI, CCR-EXPANDED, IC Timespan=2014-2020 \| \| --- \| |
| # 4 | \| AB="open science" OR TI= "open science"  Indexes=SCI-EXPANDED, SSCI, A&HCI, CPCI-S, CPCI-SSH, BKCI-S, BKCI-SSH, ESCI, CCR-EXPANDED, IC Timespan=2014-2020 \| \| --- \| |
| # 3 | \| AB="data archive" OR TI= "data archive"  Indexes=SCI-EXPANDED, SSCI, A&HCI, CPCI-S, CPCI-SSH, BKCI-S, BKCI-SSH, ESCI, CCR-EXPANDED, IC Timespan=2014-2020 \| \| --- \| |
| # 2 | \| AB="registr*" OR TI= "registr*"  Indexes=SCI-EXPANDED, SSCI, A&HCI, CPCI-S, CPCI-SSH, BKCI-S, BKCI-SSH, ESCI, CCR-EXPANDED, IC Timespan=2014-2020 \| \| --- \| |
| # 1 | \| AB="data curation" OR TI= "data curation"  Indexes=SCI-EXPANDED, SSCI, A&HCI, CPCI-S, CPCI-SSH, BKCI-S, BKCI-SSH, ESCI, CCR-EXPANDED, IC Timespan=2014-2020 \| \| --- \| |

Google Scholar

| **Search strategy** |
| --- |
| (((findable OR findability) AND (accessible OR accessibility) AND (interoperable OR interoperability) AND (reusable OR reusability)) OR "FAIR data principles" OR "FAIR guiding Principles") AND "biomedical research" AND ("data" OR metadata)  **Refined by years: 2014 - 2020** |
